# Obituaries of Female and Male Leaders From 1974 to 2016 Suggest Change in Descriptive but Stability of Prescriptive Gender Stereotypes

**DOI:** 10.3389/fpsyg.2018.02286

**Published:** 2018-11-27

**Authors:** Miriam Katharina Zehnter, Jerome Olsen, Erich Kirchler

**Affiliations:** Department of Applied Psychology, Work, Education and Economy, Faculty of Psychology, University of Vienna, Vienna, Austria

**Keywords:** gender, descriptive stereotypes, prescriptive stereotypes, leaders, obituaries, change

## Abstract

We analyzed 1415 newspaper obituaries of female and male leaders published in Germany, Austria, and Switzerland from 1974 to 2016, covering a time-span of 42 years, to investigate change in descriptive and prescriptive gender stereotypes. The obituaries’ content was condensed to four categories: *agency*, *competence*, and *communion* were used to investigate changes in descriptive stereotypes. The category *likability* was used to infer changes in prescriptive stereotypes. Consistent with theories claiming changeability of stereotypes, our results indicate changes in descriptive stereotypes. Female leaders were described as increasingly agentic over time, but not as increasingly competent. Descriptions regarding communion remained unchanged. In contrast, the description of male leaders remained relatively stable at first, followed by changes in recent years, where men were described as decreasingly competent and increasingly communal. Simultaneously, our results support theories suggesting stability of stereotypes over time indicating unchanged prescriptive stereotypes. Accordingly, increases in female leaders’ agency were associated with decreases in likability. In male leaders, increases in communion were associated with decreases in likability. Overall, our results reconcile divided theories regarding the changeability of gender stereotypes. Furthermore, our results emphasize that research and praxis need to enhance attention on prescriptive stereotypes to facilitate female leadership.

## Introduction

Gender stereotypes hinder women’s ascent to leadership. However, theory and empirical evidence regarding their changeability are divided. *Social Role Theory* (Eagly, 1987; Eagly and Wood, 2012) postulates that observing increasing numbers of women in agentic roles – e.g., leadership – changes female stereotypes toward higher agency (Koenig and Eagly, 2014). Discordantly, *Backlash Hypothesis* (Rudman and Glick, 2001; Rudman et al., 2012) questions the changeability of gender stereotypes, arguing that stereotype incongruent behavior gets punished. There is empirical support for both arguments (Diekman and Eagly, 2000; Haines et al., 2016).

Distinguishing descriptive and prescriptive stereotypes might reconcile such divided views. Descriptive stereotypes depict what men and women *are* like and lower the probability of recognizing leadership ability in women. Women stereotypically characterized as communal (e.g., kind, caring) are perceived as lacking the predominantly agentic qualities (e.g., assertive, independent) associated with successful leadership (Heilman, 1983, 2012; Eagly and Karau, 2002). However, they might change through observations of women in male domains as suggested by Social Role Theory.

Prescriptive stereotypes define what men and women *should be* like and thus exclude women from the agentic qualities demanded of leaders. Women – stereotypically required to be communal – suffered personal derogation and dislike when acting agentic (Heilman et al., 2004; Heilman and Okimoto, 2007). Thus, leadership ability in women – once recognized – is associated with high personal cost. Resonating with Backlash Hypothesis, prescriptive stereotypes might not change over time.

## Current Research

Obituaries are a unique source of information when studying stereotypes. While they are positively biased, they reveal what was appropriate to write about deceased leaders and reflect the zeitgeist of stereotypes. However, past studies that analyzed obituaries to infer changes in gender stereotypes did not distinguish between descriptive and prescriptive stereotypes (Kirchler, 1992; Rodler et al., 2001; Hartl et al., 2013). Addressing this shortcoming, we re-analyzed data of these previous studies from 1974 to 2010 with new data for 2016.

Changes in descriptive stereotypes are directly observable in obituaries as they contain information on leaders’ character and demeanor (i.e., what someone *was* like). In case of prescriptive stereotypes, however, changes are not directly observable (i.e., what someone *should* have been like). But obituaries contain evaluative information on how much leaders were liked and appreciated. Reverting to the established association between the violation of prescriptive gender stereotypes and dislike (e.g., Heilman and Okimoto, 2007), correlations of descriptions (i.e., as agentic) with evaluative information on likability (e.g., popular, esteemed) can be used to infer changes in prescriptive stereotypes.

Regarding descriptive stereotypes we hypothesized that deceased female leaders were described as increasingly agentic (e.g., assertive, independent) (H1a) and competent (e.g., experienced, expert) (H1b), but as decreasingly communal (e.g., kind, caring) over time (H1c). Concurrently, we hypothesized that increases in agency and competence of female leaders were associated with decreases in likability (e.g., popular, esteemed) (H2) expressing the assumption of stable prescriptive stereotypes. Obituaries of male leaders were included for comparison, allowing us to infer that the hypothesized changes were specific to obituaries of female leaders. Furthermore, we explored changes in obituaries of male leaders.

## Materials and Methods

### Procedure

We re-analyzed obituary data of three previously published studies that collected data from 1974 to 2010 in 6-year intervals (Kirchler, 1992; Rodler et al., 2001; Hartl et al., 2013). Furthermore, we collected data for the year 2016. The obituaries consisted of death notices of a (former) leader and a brief description of the person. A deceased person was defined as a leader when his or her leadership position in a private or public organization was explicitly named. All obituaries were written by the respective organization in which the deceased leader had been employed and only one obituary per leader was included for analysis.

To select a random sample of obituaries for analysis, a strict procedure was developed and followed throughout the years (see Kirchler, 1992). Four major daily German-language newspapers were selected (two German: *Frankfurter Allgemeine Zeitung*, *Sueddeutsche Zeitung*; one Austrian: *Die Presse*; and one Swiss: *Neue Züricher Zeitung*).

For every year, the Monday issue of the second calendar week was screened, followed by the Tuesday issue of the fourth calendar week, etc., In case no obituary was published, the issue(s) of the following day(s) were screened. In all years, the numbers of female leaders’ obituaries were small (Table 1). Therefore, a second search was conducted where *all* issues – in the relevant years – were screened. Hence, the final data consists of a random sample of male and the full population of female obituaries published in the four newspapers.

**Table 1:** Number of obituaries of female and male leaders from 1974 to 2016.

Year	Male leaders	Female leaders	Total
	(1st only)	(1st and 2nd search)	
1974	169	36 (7/29)	205
1980	127	22 (4/18)	149
1986	181	27 (9/18)	208
1992	142	26 (11/15)	168
1998	138	26 (8/18)	164
2004	90	54 (5/49)	144
2010	104	73 (11/62)	177
2016	131	69 (9/60)	200
Total	1082	333 (64/269)	1415


### Measures

All verbs, nouns, and adjectives were treated as analysis units which were assigned with codes for anonymization. Originally, Kirchler (1992) inductively derived 58 categories which all analysis units were assigned to. To better distinguish between descriptive and prescriptive stereotypes, for the present analysis, these 58 categories were categorized into four theory-based categories: *agency*, *competence*, *communion*, and *likability*.

The first three categories reflect the current state of art when categorizing gender stereotypes. Most research generally defines *agency* as stereotypically male, and *communion* as female (Ellemers, 2018). Earlier, *competence* was a component of *agency*, but recent research has derived *competence* as a separate factor (e.g., Rogers et al., 2013; Koenig and Eagly, 2014). We used agency, competence, and communion to investigate changes in descriptive gender stereotypes.

Additionally, we created the category *likability* which included words indicating how much a leader was liked and appreciated (e.g., popular, esteemed). Then, reverting to the established link between the violation of prescriptive stereotypes and dislike, we used the correlations between descriptions of deceased leaders and *likability* to infer changes in prescriptive gender stereotypes.

Six trained raters (three women and three men) conducted the categorization of the 58 previously used categories into the four categories used in the present research (Fleiss’ *κ* = 0.65)^1^. See Supplementary Table S1 of the Supplementary Material for an overview of all words categorized within the four categories agency, competence, communion, and likability, and see Supplementary Table S2 for the full data.

## Results

From 1974 to 2016, 1415 obituaries were identified for analysis. 1082 obituaries were about male and 333 about female leaders. For the latter, only 64 were found using the sampling procedure described above and 269 additional obituaries were found in the second extended search that included all issues of the respective year. As a noticeable trend, the number of obituaries dedicated to female leaders roughly doubled after the year 2000 (Table 1). An exploratory correlation between number of female obituaries and time reveals *r* = 0.82, *p* = 0.014.

### Changes in Descriptive Gender Stereotypes

**FIGURE 1:**
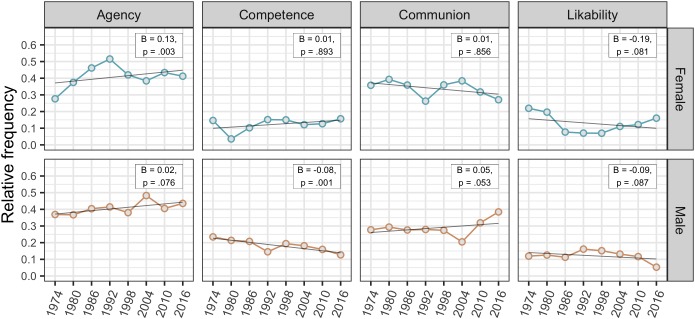
Relative frequencies of categories by gender and year. Regression estimates of absolute counts as a function of year are depicted in the top right corner.

To investigate changes in descriptive gender stereotypes, we followed two strategies. First, we describe changes in the *relative* frequencies of each category (agency, competence, and communion) used to describe female and male leaders over time (Figure 1). Second, we present negative binomial regressions for these changes calculated based on the *absolute* frequencies of each category (Supplementary Table S3). Unlike Poisson regression, negative binomial regressions can handle count data where observed variance exceeds the mean counts (over-dispersion). For each category by gender the absolute number of assignments is predicted as a function of year while controlling for the total number of category assignments. We must emphasize that due to the aggregated data the regression models are based on a very small sample and are first approximations rather than robust estimates.

#### Agency

Figure 1 shows a clear increase of agency descriptions of female leaders over time. In 1974, only 28% agentic words were used which steadily increased to 54% by 1992. After some smaller decrease, the proportion was 42% in 2016 which is 14% points higher than in 1974. This increase was confirmed by regression results. The proportion of male leaders’ agency descriptions increased slower over the years ranging between 37 and 48%. In 2016, the number of agentic words was only 7% points higher than in 1974. Regression results hinted toward a minor increase over time, but the effect was small at best. Together, these observations confirmed the assumption that the proportion of agency related words increased over time for female leaders (H1a).

#### Competence

The proportion of competence words to describe female leaders dropped from 15 to 4% from 1974 to 1980, followed by a steady increase back to 15% by 2016. Accordingly, the regression results attested no change over time. In contrast, male leaders’ competence related descriptions steadily decreased over time with 23% in 1974 and 13% in 2016. This descriptive result was confirmed by regression results. Unlike hypothesized (H1b), the percentage of competence words did not increase for female leaders. An explorative finding is that competence descriptions of male leaders decreased over time.

#### Communion

The proportions of communal descriptions of female leaders were characterized by two drops over time. Between 1974 and 1992 the percentages decreased from 36 down to 28%, followed by a rebound to 39% by 2004 and another decline to 27% by 2016. Regression results indicated no systematic change over time. Male leaders’ percentages of communion related words were steady around 28% until 1998, followed by a peaking increase in recent years to 39% by 2016. The regression results showed a small increasing trend. The observations were not in line with the assumption that female leaders are described as decreasingly communal over time (H1c). However, two decreasing developments appeared with a rebound in between.

### Changes in Prescriptive Gender Stereotypes

While descriptive gender stereotypes changed over time, prescriptive gender stereotypes could remain unchanged. One expression of this would be a negative association of women’s agency and competence with likability. Given the observed stability of competence, we limited ourselves to the relations of agency and likability.

For female leaders, the percentages of likability words were high in 1974 (22%) and 1980 (20%) but dropped in subsequent years and ranged between 7 and 12%, slightly regaining to 16% by 2016. Regression results indicated an overall decreasing trend over time. Male leaders’ likability descriptions ranged between 11 and 17% but dropped to 5% in 2016. As with female leaders, regression results hinted toward the possibility of an overall decrease.

Confirming Hypothesis 2, women’s likeability descriptions decreased as agency increased, *r* = -0.83, 95% CI [-0.97; -0.29]. In contrast, we do not find this for male leaders, *r* = -0.20, 95% CI [-0.79; 0.59]. Interestingly, explorations revealed that men’s likeability was negatively associated with communion descriptions, *r* = -0.72, 95% CI [-0.94; -0.02]. See Supplementary Table S4 of the supplement for all correlations.

## Discussion

Reconciling divided theory, we suggested that stereotypes about how women *are* might change, but stereotypes about how women *should be* might not. Results from the (re-)analysis of newspaper obituaries dedicated to female and male leaders of private and public organizations in Germany, Austria, and Switzerland supported this notion. Consistent with theories claiming changeability of stereotypes (e.g., Social Role Theory), female leaders were described as increasingly agentic over time; however, not as increasingly competent. Female leaders were not described as decreasingly communal over time. In contrast, male leaders were described as decreasingly competent, and in recent years as increasingly communal. Overall, these results indicate change in descriptive gender stereotypes.

Simultaneously, our results supported theories assuming stability of stereotypes over time (e.g., Backlash Hypothesis). Increased proportions of agency words were associated with decreased proportions of likability words in obituaries of female leaders. Furthermore, explorations revealed that proportions of communion words and likability words correlated negatively in obituaries of male leaders. Reverting to the established association between gendered prescriptions and (dis)likeability (e.g., Heilman and Okimoto, 2007), we concluded that prescriptive gender stereotypes remained unchanged over time.

Alternatively, a general decline of trust in leadership might explain decreases in likability of leaders over time. But opinion polls e.g., in Germany, showed overall high levels of trust in leaders (Nink, 2014; Haufe Akademie, 2015). Moreover, in this case, we should have found *positive* relations between communion and likability. After all, trust was linked to “feminine” leadership styles (Dirks and Ferrin, 2002; Eagly et al., 2003). In our data, however, communion and likability were unrelated for female leaders, and negatively related for male leaders.

Ultimately, our results raise questions about change mechanisms of prescriptive stereotypes which might be quantitatively different from those of descriptive gender stereotypes. The observation of women in agentic roles might lead to slower changes in prescriptive stereotypes indicating more pronounced “cultural lag” (Diekman et al., 2010). However, change mechanisms might differ qualitatively with prescriptive gender stereotypes following different change mechanisms altogether. Future research should consistently distinguish between descriptive and prescriptive stereotypes and clarify questions in this regard.

One limitation of this study was the re-analysis of previously aggregated data. Individual data would have allowed more elaborate and robust inferential analysis. Strengths of the present study were the long time-span observed and using unobtrusive measures. In opinion polls and questionnaire studies, research participants’ awareness of being observed might alter response-behavior (Podsakoff et al., 2003). Unobtrusive methods are therefore particularly suited for the research of sensitive issues such as stereotypes.

Last, our results implicate that merely increasing numbers of female leaders (i.e., by quotas), although important, might be insufficient. To support female leadership permanently, understanding and breaking prescriptive norms for women and men is essential.

## Data Availability

All datasets generated and analyzed for this study are included in the manuscript and the Supplementary Files.

## Author Contributions

EK und MZ contributed to the conception and design of the study. EK and JO organized and supervised data collection. JO performed the statistical analysis. MZ wrote the first draft of the manuscript. JO wrote sections of the manuscript. All authors contributed to manuscript revision, read, and approved the submitted version.

## Conflict of Interest Statement

The authors declare that the research was conducted in the absence of any commercial or financial relationships that could be construed as a potential conflict of interest.

## Footnotes

1 To follow the exact same procedure for all years, analysis units for 2016 were also first assigned to the 58 content-categories and then to the four new categories.

## References

[B1] Diekman A. B. Eagly A. H. (2000). Stereotypes as dynamic constructs: women and men of the past, present, and future. *Pers. Soc. Psychol. Bull.* 26 1171–1188. 10.1177/0146167200262001

[B2] Diekman A. B. Eagly A. H. Johnston A. M. (2010). “Social structure,” in *The SAGE Handbook of Prejudice, Stereotyping and Discrimination*, eds Dovidio J. F. Hewstone M. Glick P. Esses V. M. (London: SAGE Publications Ltd.), 209–224. 10.4135/9781446200919.n13

[B3] Dirks K. T. Ferrin D. L. (2002). Trust in leadership: meta-analytic findings and implications for research and practice. *J. Appl. Psychol.* 87 611–628. 10.1037//0021-9010.87.4.611 12184567

[B4] Eagly A. H. (1987). *Sex Differences in Social Behavior: A Social-Role Interpretation.* Hillsdale, NJ: Erlbaum.

[B5] Eagly A. H. Johannesen-Schmidt M. C. van Engen M. L. (2003). Transformational, transactional, and laissez-fair leadership styles: a meta-analysis comparing women and men. *Psychol. Bull.* 129 569–591. 10.1037/0033-2909.129.4.569 12848221

[B6] Eagly A. H. Karau S. J. (2002). Role congruity theory of prejudice toward female leaders. *Psychol. Rev.* 109 573–598. 10.1037/0033-295X.109.3.573 12088246

[B7] Eagly A. H. Wood W. (2012). “Social role theory,” in *Handbook of Theories of Social Psychology*, eds Van Lange P. A. M. Kruglanski A. W. Higgins E. T. (Thousand Oaks, CA: SAGE Publications Ltd.), 458–476. 10.4135/9781446249222.n49

[B8] Ellemers N. (2018). Gender stereotypes. *Annu. Rev. Psychol.* 69 275–298. 10.1146/annurev-psych-122216-011719 28961059

[B9] Haines E. L. Deaux K. Lofaro N. (2016). The times they are a-changing… or are they not? A comparison of gender stereotypes, 1983–2014. *Psychol. Women Q.* 40 353–363. 10.1177/0361684316634081

[B10] Hartl B. Kirchler E. Muehlbacher S. (2013). Geschlechterstereotype auf führungsebene zwischen 1974 und 2010. *Z. Arbeits Organ.* 57 121–131. 10.1026/0932-4089/a000114

[B11] Haufe Akademie. (2015). *Repräsentative Forsa-Umfrage. Vertrauen in Führungskräfte und Unternehmen.* Available at: https://www.haufe-akademie.de/downloadserver/Perspektiven-Blog/Umfragedaten_HaufeAkademie_forsa_Wasistrichtig.pdf

[B12] Heilman M. E. (1983). Sex bias in work settings: the lack of fit model. *Res. Organ. Behav.* 5 269–298.

[B13] Heilman M. E. (2012). Gender stereotypes and workplace bias. *Res. Organ. Behav.* 32 113–135. 10.1016/j.riob.2012.11.003

[B14] Heilman M. E. Okimoto T. G. (2007). Why are women penalized for success at male tasks? The implied communality deficit. *J. Appl. Psychol.* 92 81–92. 10.1037/0021-9010.92.1.81 17227153

[B15] Heilman M. E. Wallen A. S. Fuchs D. Tamkins M. M. (2004). Penalties for success: reactions to women who succeed at male gender-typed tasks. *J. Appl. Psychol.* 89 416–427. 10.1037/0021-9010.89.3.416 15161402

[B16] Kirchler E. (1992). Adorable woman, expert man: changing gender images of women and men in management. *Eur. J. Soc. Psychol.* 22 363–373. 10.1002/ejsp.2420220405

[B17] Koenig A. M. Eagly A. H. (2014). Evidence for the social role theory of stereotype content: observations of groups’ roles shape stereotypes. *J. Pers. Soc. Psychol.* 107 371–392. 10.1037/a0037215 25133722

[B18] Nink M. (2014). *Engagement Index. Die Neuesten Daten und Erkenntnisse aus 13 Jahren Gallup-Studie.* Munich: Redline Verlag.

[B19] Podsakoff P. M. MacKenzie S. B. Lee J.-Y. Podsakoff N. P. (2003). Common method biases in behavioral research: a critical review of the literature and recommended remedies. *J. Appl. Psychol.* 88 879–903. 10.1037/0021-9010.88.5.879 14516251

[B20] Rodler C. Kirchler E. Hoezl E. (2001). Gender stereotypes of leaders: an analysis of the contents of obituaries from 1974 to 1998. *Sex Roles* 45 827–843. 10.1023/A:1015644520770

[B21] Rogers K. B. Schröder T. Scholl W. (2013). The affective structure of stereotype content: behavior and emotion in intergroup context. *Soc. Psychol. Q.* 76 125–150. 10.1177/0190272513480191

[B22] Rudman L. A. Glick P. (2001). Prescriptive gender stereotypes and backlash toward agentic women. *J. Soc. Issues* 57 743–762. 10.1111/0022-4537.00239

[B23] Rudman L. A. Moss-Racusin C. A. Phelan J. E. Nauts S. (2012). Status incongruity and backlash effects: defending the gender hierarchy motivates prejudice against female leaders. *J. Exp. Soc. Psychol.* 48 165–179. 10.1016/j.jesp.2011.10.008

