# Supplementary material for: Obituaries of Female and Male Leaders From 1974 to 2016 Suggest Change in Descriptive but Stability of Prescriptive Gender Stereotypes
Source: Front Psychol. 2018 Nov 27;9:2286. doi: 10.3389/fpsyg.2018.02286 (PMC6277582; doi:10.3389/fpsyg.2018.02286)
Supplement: Supplementary file 1 [file Table_1.docx]

***Supplementary Material***

**Obituaries of Female and Male Leaders from 1974 to 2016 Suggest Change in Descriptive but Stability of Prescriptive Gender Stereotypes**

**Miriam Katharina Zehnter*, Jerome Olsen, Erich Kirchler**

***Correspondence: Miriam Katharina Zehnter:** [**miriam.zehnter@univie.ac.at**](mailto:miriam.zehnter@univie.ac.at)

| Table S1. Further categorization of the 58 original categories to the four categories used in the present study. | | | |
| --- | --- | --- | --- |
| *Agency* | *Competence* | *Communion* | *Likability* |
| Active | Able | Balanced | Appreciated |
| Committed | Accurate | Benevolent | Deserving |
| Convincing | Creative | Calm | Esteemed |
| Courageous | Experienced | Caring | Honored |
| Decision maker | Expert | Companionate | Popular |
| Diligent | Intelligent | Considerate | Venerable |
| Entrepreneurship | Professional | Dutiful |  |
| Far-sighted | Successful | Fair |  |
| Indefatigable |  | Friendly |  |
| Independent |  | Helpful |  |
| Initiator |  | Honest |  |
| Leader |  | Humane |  |
| Opinion maker |  | Humble |  |
| Organizer |  | Kind |  |
| Personality |  | Loyal |  |
| Pioneer |  | Open-minded |  |
| Strong |  | Ready to compromise |  |
| Teacher |  | Reliable |  |
| Work-oriented |  | Responsible |  |
|  |  | Servant |  |
|  |  | Sociable |  |
|  |  | Unselfish |  |

| *Note*. Three original categories could not be categorized further (patriotic, religious, animal/nature protection advocate). |
| --- |
