# Supplementary material for: Obituaries of Female and Male Leaders From 1974 to 2016 Suggest Change in Descriptive but Stability of Prescriptive Gender Stereotypes
Source: Front Psychol. 2018 Nov 27;9:2286. doi: 10.3389/fpsyg.2018.02286 (PMC6277582; doi:10.3389/fpsyg.2018.02286)
Supplement: Supplementary file 2 [file Table_2.docx]

***Supplementary Material***

**Obituaries of Female and Male Leaders from 1974 to 2016 Suggest Change in Descriptive but Stability of Prescriptive Gender Stereotypes**

**Miriam Katharina Zehnter*, Jerome Olsen, Erich Kirchler**

***Correspondence: Miriam Katharina Zehnter: miriam.zehnter@univie.ac.at**

| Table S2. Raw data frame. | | | | | | | | | | | | | | | |  |
| --- | --- | --- | --- | --- | --- | --- | --- | --- | --- | --- | --- | --- | --- | --- | --- | --- |
|  |  |  | Agency | |  | Competence | |  | Communion | |  | Likeability | |  |  |  |
| Gender | Year |  | *f* | *f*_relative_ |  | *f* | *f*_relative_ |  | *f* | *f*_relative_ |  | *f* | *f*_relative_ |  | Total number  of assignments | Number of  obituaries |
| female | 1974 |  | 34 | 0.28 |  | 18 | 0.15 |  | 44 | 0.36 |  | 27 | 0.22 |  | 123 | 36 |
| female | 1980 |  | 21 | 0.38 |  | 2 | 0.04 |  | 22 | 0.39 |  | 11 | 0.20 |  | 56 | 22 |
| female | 1986 |  | 36 | 0.46 |  | 8 | 0.10 |  | 28 | 0.36 |  | 6 | 0.08 |  | 78 | 27 |
| female | 1992 |  | 51 | 0.54 |  | 10 | 0.11 |  | 26 | 0.28 |  | 7 | 0.07 |  | 94 | 26 |
| female | 1998 |  | 42 | 0.43 |  | 13 | 0.13 |  | 36 | 0.37 |  | 7 | 0.07 |  | 98 | 26 |
| female | 2004 |  | 73 | 0.39 |  | 19 | 0.10 |  | 73 | 0.39 |  | 21 | 0.11 |  | 186 | 54 |
| female | 2010 |  | 82 | 0.44 |  | 22 | 0.12 |  | 60 | 0.32 |  | 23 | 0.12 |  | 187 | 73 |
| female | 2016 |  | 108 | 0.42 |  | 39 | 0.15 |  | 71 | 0.27 |  | 42 | 0.16 |  | 260 | 69 |
| male | 1974 |  | 269 | 0.37 |  | 168 | 0.23 |  | 202 | 0.28 |  | 87 | 0.12 |  | 726 | 169 |
| male | 1980 |  | 184 | 0.37 |  | 105 | 0.21 |  | 147 | 0.29 |  | 63 | 0.13 |  | 499 | 127 |
| male | 1986 |  | 313 | 0.41 |  | 156 | 0.20 |  | 214 | 0.28 |  | 87 | 0.11 |  | 770 | 181 |
| male | 1992 |  | 257 | 0.43 |  | 69 | 0.12 |  | 173 | 0.29 |  | 100 | 0.17 |  | 599 | 142 |
| male | 1998 |  | 233 | 0.40 |  | 91 | 0.16 |  | 168 | 0.29 |  | 93 | 0.16 |  | 585 | 138 |
| male | 2004 |  | 165 | 0.48 |  | 61 | 0.18 |  | 70 | 0.21 |  | 45 | 0.13 |  | 341 | 90 |
| male | 2010 |  | 160 | 0.41 |  | 60 | 0.15 |  | 126 | 0.32 |  | 46 | 0.12 |  | 392 | 104 |
| male | 2016 |  | 230 | 0.44 |  | 66 | 0.13 |  | 203 | 0.39 |  | 28 | 0.05 |  | 527 | 131 |
| *Note.* Each obituary could be assigned to multiple categories. Therefore, the table distinguished between total number of assignments and total number of obituaries in a given year. | | | | | | | | | | | | | | | | |
