# Supplementary material for: Obituaries of Female and Male Leaders From 1974 to 2016 Suggest Change in Descriptive but Stability of Prescriptive Gender Stereotypes
Source: Front Psychol. 2018 Nov 27;9:2286. doi: 10.3389/fpsyg.2018.02286 (PMC6277582; doi:10.3389/fpsyg.2018.02286)
Supplement: Supplementary file 3 [file Table_3.docx]

***Supplementary Material***

**Obituaries of Female and Male Leaders from 1974 to 2016 Suggest Change in Descriptive but Stability of Prescriptive Gender Stereotypes**

**Miriam Katharina Zehnter*, Jerome Olsen, Erich Kirchler**

***Correspondence: Miriam Katharina Zehnter:** [**miriam.zehnter@univie.ac.at**](mailto:miriam.zehnter@univie.ac.at)

| Table S3. Negative binomial regressions for each gender predicting the number of analysis units assigned to a respective category over time. | | | | | | | | | | | | | | | | | |
| --- | --- | --- | --- | --- | --- | --- | --- | --- | --- | --- | --- | --- | --- | --- | --- | --- | --- |
|  |  | Agency | | | | | | | | | | | | |  | |  |
|  |  | Male | | | | |  | | Female | | | | | |  | |  |
|  |  | *B* | *SE* | | *p* | |  | | *B* | | *SE* | | *p* | |  | |  |
| Intercept |  | 4.11 | 0.20 | | < .001 | |  | | 2.90 | | 0.14 | | < .001 | |  | |  |
| Year |  | 0.02 | 0.01 | | .076 | |  | | 0.13 | | 0.04 | | .003 | |  | |  |
| Num. Assignments |  | 0.01 | 0.00 | | < .001 | |  | | 0.01 | | 0.00 | | .034 | |  | |  |
|  |  | Competence | | | | | | | | | | | | |  | |  |
|  |  | Male | | | | |  | | Female | | | | | |  | |  |
|  |  | *B* | *SE* | | *p* | |  | | *B* | | *SE* | | *p* | |  | |  |
| Intercept |  | 3.81 | 0.37 | | < .001 | |  | | 1.44 | | 0.36 | | < .001 | |  | |  |
| Year |  | -0.08 | 0.03 | | .001 | |  | | 0.01 | | 0.11 | | .893 | |  | |  |
| Num. Assignments |  | 0.01 | 0.00 | | < .001 | |  | | 0.03 | | 0.01 | | .038 | |  | |  |
|  |  | Communion | | | | | | | | | | | | |  | |  |
|  |  | Male | | | | |  | | Female | | | | | |  | |  |
|  |  | *B* | *SE* | | *p* | |  | | *B* | | *SE* | | *p* | |  | |  |
| Intercept |  | 3.15 | 0.40 | | < .001 | |  | | 2.90 | | 0.21 | | < .001 | |  | |  |
| Year |  | 0.05 | 0.03 | | .053 | |  | | 0.01 | | 0.06 | | .856 | |  | |  |
| Num. Assignments |  | 0.01 | 0.00 | | < .001 | |  | | 0.02 | | 0.01 | | .017 | |  | |  |
|  |  | Likability | | | | | | | | | | | | | |  | |
|  |  | Male | | | | | |  | | Female | | | | | |  | |
|  |  | *B* | | *SE* | | *p* | |  | | *B* | | *SE* | | *p* | |  | |
| Intercept |  | 4.03 | | 0.78 | | < .001 | |  | | 1.53 | | 0.37 | | < .001 | |  | |
| Year |  | -0.09 | | 0.05 | | .087 | |  | | -0.19 | | 0.11 | | .081 | |  | |
| Num. Assignments |  | 0.00 | | 0.00 | | .330 | |  | | 0.05 | | 0.01 | | < .001 | |  | |

*Note*. *N* = 8.
