# Supplementary material for: Obituaries of Female and Male Leaders From 1974 to 2016 Suggest Change in Descriptive but Stability of Prescriptive Gender Stereotypes
Source: Front Psychol. 2018 Nov 27;9:2286. doi: 10.3389/fpsyg.2018.02286 (PMC6277582; doi:10.3389/fpsyg.2018.02286)
Supplement: Supplementary file 4 [file Table_4.docx]

***Supplementary Material***

**Obituaries of Female and Male Leaders from 1974 to 2016 Suggest Change in Descriptive but Stability of Prescriptive Gender Stereotypes**

**Miriam Katharina Zehnter*, Jerome Olsen, Erich Kirchler**

***Correspondence: Miriam Katharina Zehnter: miriam.zehnter@univie.ac.at**

| Table S4. Correlations with 95% confidence intervals between agency, competence, communion, and likability in obituaries for female and male leaders | | | | |
| --- | --- | --- | --- | --- |
|  | Agency | Competence | Communion | Likability |
| Agency | 1 | .12  [-.64, .76] | -.54  [-.90, .27] | -.83  [-.97, -.29] |
| Competence | -.59  [-.91, .24] | 1 | -.63  [-.93, .13] | -.30  [-.83, .51] |
| Communion | -.23  [-.80, .57] | -.50  [-.89, .31] | 1 | .23  [-.56, .81] |
| Likability | -.20  [-.79, .59] | .31  [-.51, .83] | -.72  [-.94, -.02] | 1 |
| *Note.* Correlation coefficients for female leaders are displayed above, and for male leaders below the diagonal | | | | |
